# Broadly sampled orthologous groups of eukaryotic proteins for the phylogenetic study of plastid-bearing lineages

**DOI:** 10.1186/s13104-021-05553-4

**Published:** 2021-04-17

**Authors:** Mick Van Vlierberghe, Hervé Philippe, Denis Baurain

**Affiliations:** 1 InBioS – PhytoSYSTEMS, Eukaryotic Phylogenomics, University of Liège, Liège, Belgium; 2 Station D’Ecologie Théorique Et Expérimentale de Moulis, UMR CNRS 5321, Moulis, France; 3 Département de Biochimie, Centre Robert-Cedergren, Université de Montréal, Montréal, Québec Canada

**Keywords:** Orthology, Phylogenomics, Algae, CASH, Proteomes, Eukaryotic evolution, Contamination, Organelles, Endosymbiotic gene transfer (EGT), Horizontal or lateral gene transfer (HGT/LGT), Kleptoplasty

## Abstract

**Objectives:**

Identifying orthology relationships among sequences is essential to understand evolution, diversity of life and ancestry among organisms. To build alignments of orthologous sequences, phylogenomic pipelines often start with all-vs-all similarity searches, followed by a clustering step. For the protein clusters (orthogroups) to be as accurate as possible, proteomes of good quality are needed. Here, our objective is to assemble a data set especially suited for the phylogenomic study of algae and formerly photosynthetic eukaryotes, which implies the proper integration of organellar data, to enable distinguishing between several copies of one gene (paralogs), taking into account their cellular compartment, if necessary.

**Data description:**

We submitted 73 top-quality and taxonomically diverse proteomes to OrthoFinder. We obtained 47,266 orthogroups and identified 11,775 orthogroups with at least two algae. Whenever possible, sequences were functionally annotated with eggNOG and tagged after their genomic and target compartment(s). Then we aligned and computed phylogenetic trees for the orthogroups with IQ-TREE. Finally, these trees were further processed by identifying and pruning the subtrees exclusively composed of plastid-bearing organisms to yield a set of 31,784 clans suitable for studying photosynthetic organism genome evolution.

## Objective

Our main objective is to analyse the phylogenetic origin of plastid-targeted genes in complex algae [1–3] in a fully automated fashion. To do so, we designed and developed a series of strategies and tools around a large-scale single-gene tree analysis pipeline. The first step was to build alignments of orthologous sequences with OrthoFinder, a high accuracy orthogroup inference algorithm [4]. We focused on top-quality proteomes, especially with high completeness, which is essential to obtain the most complete and balanced OGs possible [5, 6]. In order to maximize completeness and to facilitate the phylogenetic analysis, we complemented beforehand the proteomes having no or only incomplete plastid and/or nucleomorph sequences. Then we processed the resulting OGs, first by isolating the OGs containing photosynthetic organisms, and second by sorting out gene copies shared by plastid-bearing algae from their paralogs. To this end, we built trees using IQ-TREE [7] and used our own tool (tree-clan-splitter.pl) to detect and prune the subtree(s) of interest.

## Data description

We collected 73 top-quality eukaryotic proteomes (i.e., conceptually translated genomes; Data file 1, Data set 1, Data set 2) with high completeness (Data file 2) [5, 6] and low contamination levels (Data set 3) [8, 9] (Table 1). Those were selected to be taxonomically diverse, covering all photosynthetic phyla [10, 11], along with some non-photosynthetic organisms to be used as beacons by our clan-identifying algorithm. Those proteomes were complemented with organellar (i.e., plastid and nucleomorph) proteins if they were partly or fully missing in the original source. Hence, 16 were complemented with plastid proteomes whereas two were complemented with nucleomorph proteomes. All proteomes (complemented or not) were dereplicated with CD-HIT [12]. In addition, we used tag-loc-ids.pl, a custom tool designed to tag sequence identifiers according to their encoding genome and cellular localization, such as nuclear-encoded-and-plastid-targeted (nucpt#), nuclear-encoded-periplastid-compartment-targeted (nuppct#), plastid-encoded-plastid-targeted (cpcpt#), nucleomorph-encoded (nm#), and mitochondrion-encoded (mt#), to facilitate subsequent phylogenetic analyses. Then, we used OrthoFinder [4] for orthology inference, which resulted in 47,266 OGs (Data file 3, Data set 4), composed of two or more sequences belonging to eleven main taxonomic groups (according to NCBI Taxonomy [13]), either classified as “primary algae” (Glaucocystophyceae, Rhodophyta, Viridiplantae) or “complex algae” (Apicomplexa, Colpodellida, Dinophyceae, Cryptophyceae, Euglenozoa, Ochrophyta (including Pelagophyceae), Haptophyta, and Chlorarachniophyceae). Hence, OGs were tabulated into three different categories: “two-algae” (at least one complex alga from two different groups or at least one complex alga and one primary alga, n = 11,775), “one-alga” (at least one alga, n = 18,844) and “zero-algae” (no algae, n = 16,647) using the script classify-mcl-out.pl. In order to address the issue of multiple-copy genes (paralogs), we developed a strategy to isolate subtrees (“clans”) of interest, i.e., including only plastid-bearing organisms. Briefly, we computed trees for the 11,775 “two-algae” OGs when possible (i.e., ≥ 3 sequences, n = 11,499) with IQ-TREE [7] and developed a tool for identifying and pruning subtrees fulfilling user-specified taxonomic filters (tree-clan-splitter.pl). This way, we obtained 31,784 “photosynthetic” clans (Data set 5) only composed of plastid-bearing organisms (including species with a non-photosynthetic plastid, such as *Plasmodium falciparum*). Additionally, we provide detailed annotation reports obtained with eggNOG [14].

**Table 1:** Overview of data files/data sets

Label	Name of data file/data set	File types (file extension)	Data repository and identifier (DOI or accession number)
Additional file 1	Methods	PDF file (.pdf)	Figshare https://doi.org/10.6084/m9.figshare.13604102.v3 [18]
Data file 1	Taxonomic sampling	Image file (.png)	Figshare https://doi.org/10.6084/m9.figshare.13603511.v1 [19]
Data set 1	Proteome set description	Text files (.csv,.html)	Figshare https://doi.org/10.6084/m9.figshare.13113893.v1 [20]
Data set 2	Proteome files	FASTA files (.tar.gz)	Figshare https://doi.org/10.6084/m9.figshare.13573424.v2 [21]
Data file 2	BUSCO report	Text file (.csv)	Figshare https://doi.org/10.6084/m9.figshare.13235045.v1 [22]
Data set 3	Forty-Two reports and configuration files	Text files (.tsv,.csv,.yaml)	Figshare https://doi.org/10.6084/m9.figshare.13235063.v3 [23]
Data file 3	Orthogroup properties	Image file (.pdf)	Figshare https://doi.org/10.6084/m9.figshare.13312622.v1 [24]
Data set 4	Orthogroups	FASTA files, YAML configuration file (.tar.gz)	Figshare https://doi.org/10.6084/m9.figshare.13573658.v3 [25]
Data set 5	Clans	FASTA files (.tar.gz)	Figshare https://doi.org/10.6084/m9.figshare.13573415.v1 [26]
Data file 4	Organelle database	Text file (.tsv)	Figshare https://doi.org/10.6084/m9.figshare.13246841.v1 [27]
Data file 5	Plastid-targeted proteins	Spreadsheet (.xlsx)	Figshare https://doi.org/10.6084/m9.figshare.13246784.v1 [28]
Data file 6	eggNOG OG annotations	Text file (.tsv)	Figshare https://doi.org/10.6084/m9.figshare.13415048.v1 [29]
Data file 7	eggNOG clan annotations	Text file (.tsv)	Figshare https://doi.org/10.6084/m9.figshare.13415060.v1 [30]

## Limitations

- Occasionally, organellar genome sequences are from a different strain than the nucleus data; it could be an issue if we were trying to resolve relationships between close relatives of the same lineage. Nonetheless, it is not the case here, since the major endosymbiotic-like events we are tracking occurred most certainly between distinct lineages.
- The way we handle the tagging overwrites the information about potential NUMTs, NUNMs and NUPTs; this means that if a gene existed in both genomic compartments (nucleus and organelle) we always retained the organellar counterpart.
- Only a few of the nucleus-encoded-and-plastid-targeted proteins (nucpt#) were identified by proteomics (e.g., in *P. falciparum)* [17]; the remaining are the results of in silico predictions [15, 16], which are less reliable than proteomic experiments.

## Data Availability

All data generated or analysed during this study are publicly available in the figshare repository (https://doi.org/10.6084/m9.figshare.13604102.v3, https://doi.org/10.6084/m9.figshare.13603511.v1, https://doi.org/10.6084/m9.figshare.13113893.v1, https://doi.org/10.6084/m9.figshare.13573424.v2, https://doi.org/10.6084/m9.figshare.13235045.v1, https://doi.org/10.6084/m9.figshare.13235063.v3, https://doi.org/10.6084/m9.figshare.13312622.v1, https://doi.org/10.6084/m9.figshare.13573658.v3, https://doi.org/10.6084/m9.figshare.13573415.v1, https://doi.org/10.6084/m9.figshare.13246841.v1, https://doi.org/10.6084/m9.figshare.13246784.v1, https://doi.org/10.6084/m9.figshare.13415048.v1, https://doi.org/10.6084/m9.figshare.13415060.v1). Please see Table 1 and reference list [18–30] for details and links to the data.

## Abbreviations

OGs: Orthologous groups or orthogroups
NUMTs: Nuclear mitochondrial DNAs
NUNMs: Nucleomorph-derived DNAs
NUPTs: Nuclear plastid DNAs
